# Working experiences of remote interpreters in health care settings—insights from Austria and Germany

**DOI:** 10.3389/fpubh.2025.1477965

**Published:** 2025-02-10

**Authors:** Sophie Klomfar, Anna Teufel, Gernot Gerger, Maria Kletečka-Pulker, Klara Doppler, Magdalena Eitenberger, Sabine Völkl-Kernstock

**Affiliations:** ^1^Ludwig Boltzmann Institute Digital Health and Patient Safety, Medical University of Vienna, Vienna, Austria; ^2^Clinical Department of Pediatric Pulmonology, Allergology and Endocrinology, University Clinic for Pediatrics and Adolescent Medicine, Medical University of Vienna, Vienna, Austria; ^3^Institute for Ethics and Law in Medicine, University of Vienna, Vienna, Austria; ^4^Institute for Political Science, Faculty of Social Sciences, University of Vienna, Vienna, Austria; ^5^Department of Child and Adolescent Psychiatry, Medical University of Vienna, Vienna, Austria

**Keywords:** video remote interpretation, medical interpreting, stress, job dissatisfaction, language barrier, quantitative methods, qualitative methods

## Abstract

**Background:**

The rise in linguistically diverse patient populations has introduced significant challenges in healthcare due to language barriers. Video Remote Interpreting (VRI) has emerged as a cost-effective solution in healthcare settings. However, its impact on interpreters, particularly the specific enabling and hindering factors from their point of view remains underexplored. For example, in some studies, VRI interpreters report higher stress and job dissatisfaction. We hypothesize that interpreters’ work experience and supervision attendance mitigate negative effects. We tested this hypothesis using a quantitative approach. Additionally, we analyzed qualitative data to uncover more enabling and hindering factors.

**Methods:**

A sample of 87 interpreters working in Austria and Germany was included in this multi-methods study. Stress, job dissatisfaction, work experience, and supervision were analyzed using correlations and group comparisons. Responses to open-ended questions were analyzed using thematic content analysis to identify enabling and hindering factors, with network analysis exploring their interconnections.

**Results:**

Longer work experience correlated with lower stress. Supervision had no significant effect on stress or job satisfaction. Thematic content analysis identified 21 factors affecting VRI: While VRI enhances efficiency and emotional distance, interpreters face technical problems and difficulties arising from the lack of physical presence. Network analysis confirmed that VRI settings are characterized by a close interplay between these enabling and hindering factors.

**Discussion:**

Strategies for using VRI can be derived from these data. VRI is an efficient alternative to in-person interpreting, with challenges that can be mitigated. Training healthcare personnel in handling VRI and optimizing VRI conditions can contribute to better healthcare outcomes.

## 1 Introduction

In recent years, the healthcare sector in Austria and many other countries has seen a surge in challenges caused by language barriers, the outcome of increasingly linguistically diverse patient populations. This requires quick, practical, reliable, and safe solutions which consider the specific interests of all parties involved.

Language barriers pose a major challenge for healthcare professionals and institutions. They impact a large majority of physicians in Austrian hospitals, multiple times a week if not daily (1). In the context of communication barriers, it is imperative to acknowledge that the paradigm shift toward patient empowerment and autonomy—superseding paternalistic care models—depends on clear and comprehensible patient-physician communication on a partnership basis (2, 3). Not only can the incomplete and incomprehensible exchange of relevant medical information make it impossible to establish informed consent, thereby posing a significant liability risk (4–7), but it also negatively affects healthcare professionals’ work routines and satisfaction (1). Besides gravely impacting health care professionals’ work routine, patients with limited language proficiency may experience myriad healthcare disparities associated with language barriers. These may include increased difficulty in accessing health care and preventive measures (8–11), as well as numerous problems during treatment and diagnosis (12, 13) including longer hospitalizations, poorer adherence to treatment regimens and follow ups, more medication complications, and decreased patient satisfaction (14). Thus, overcoming language barriers and guaranteeing clear and comprehensible communication in every step of the treatment process is an essential prerequisite in every treatment, and supports patient and staff safety.

As a result various interventions have been introduced in the healthcare sector with the objective of serving linguistically diverse patient populations and preventing negative consequences associated with language barriers. Commonly, two interpreter strategies are employed, differing significantly in terms of quality. One approach utilizes the services of lay persons without formal training, whereas the other relies on the expertise of professional interpreters, who provide on-site in-person or remote support through modalities such as video remote interpreting (VRI) or telephone remote interpreting (TRI). The utilization of lay interpreters is associated with several issues, including the potentially unethical deployment of language-proficient family members or other lay persons and significant challenges in the quality and reliability of interpretation (15).

In contrast, VRI represents a cost effective, reliable and secure method for providing professional interpretation services (9, 16). In some cases, in-person settings are preferable over VRI settings due to factors such as the desire to establish better rapport, personal preference of patients, complexity of the medical procedure, trust, or technical competency and possibilities. However, research has demonstrated that in general VRI and TRI have been shown to achieve clinician and patient satisfaction levels comparable to those achieved through in-person interpreting (17–21). VRI ensures reliable and satisfactory interpretation outcomes, thus preventing liability risks due to interpreter errors (22–24) and furthering patient safety (17).

In Austria, in 2013, a new professional VRI system with safe online connections was implemented in various clinical settings as part of a pilot project on quality assurance in the treatment of non-German speaking patients. Following positive feedback from both physicians and patients (1), remote interpreting in audio and video settings for various applications (health, social services, legal) is now an established service provided by *SAVD Videodolmetschen GmbH*, a specialist audio and video dialogue service provider (25).

The role of the interpreter is more than simply to translate a dialogue. Whenever interpreting services are used, it is important to bear in mind that the patient-physician conversation, usually characterized by a dialogue between two people, involves a third party: the interpreter. The medical interpreter’s main task lies in translating the information, in this case the words spoken by the physician and patient. However, they often find themselves in situations in which they are called upon to mediate and negotiate between medical professionals and patients, while simultaneously accounting for potential cultural differences and various socio-contextual factors. Thus, medical interpreters often find themselves subject to a range of different expectations, requiring them to quickly adapt to the specific circumstances, and having to manage conflicting roles, which may change within the interpretation process (26–28). For example, an interpreter may not be able to retain their neutrality when the situation demands they show empathy toward the client (14). Critically, to date, the role of the interpreter in the remote interpretation setting has not yet been sufficiently subjected to scientific analysis. VRI is increasingly gaining acceptance due to its many positive characteristics, making it essential to address the role of the medical interpreter in this specific remote setting.

Studies on VRI in the public health sector have primarily focused on the satisfaction of healthcare professionals and patients. While both healthcare professionals and patients appear satisfied with VRI, some studies show that interpreters prefer to work in an in-person setting (20, 29, 30). It is notable that recent studies fail to address the perceptions and satisfaction of video remote interpreters themselves, especially considering the surge in remote interpreting since the onset of the COVID-19 pandemic (31, 32) and the growth in technical solutions for VRI over the last two decades. There is a large and growing demand for interpreters willing to work with VRI or TRI (33), and therefore it is important to collect and analyze VRI-interpreter experiences. This can help create awareness of enabling factors, reduce possible associated challenges, and thus improve working conditions for video interpreters. Regarding the perception of stress in VRI in non-medical settings, VRI interpreters who provide simultaneous interpretations at conferences report higher subjective levels of stress (34). The aspect of experienced stress is important to consider in non-medical settings as previous research shows that work-related stress, poor wellbeing (i.e., job satisfaction), and moderate-to-high levels of burnout among healthcare professionals are associated with poorer patient safety outcomes and increased medical errors (35–37). Although medical video interpreters are not healthcare workers, as they are required to cooperate closely with healthcare professionals, patient and staff safety could be compromised by their work-related stress and job dissatisfaction and these factors will therefore be included in this study.

Having work experience with VRI settings could attenuate these negative effects. Healthcare professionals show more satisfaction with VRI the longer it is used (38). Therefore, it is of interest to examine, whether similar trends apply in interpreters, potentially influencing interpreters’ levels of satisfaction and subjective stress levels when using VRI. Thus, work experience was included in this study.

Supervision could also be an effective means of reducing work-related stress and increasing satisfaction, also in the interpreting profession (39). Supervision is a professional consulting method focused on addressing work-related challenges faced by individuals, teams, groups, or organizations to enable participants to deal constructively with the associated problems and challenging or stressful work situations. Under the guidance by a supervisor, participants reflect on work-related issues and develop alternative approaches for the future (40). The opportunity for interpreters to reflect on their own work within a secure environment has the potential to enhance not only their mental well-being but also the safety of the individuals they serve, consequently also reinforcing patient safety. Therefore, one of our main questions was whether supervision affected chronic stress or job dissatisfaction levels. Gender might be an additional moderating factor in this respect. For example, there are reports that women experience higher levels of stress in the workplace, however, the evidence is mixed (41). Further, participation in supervision may be hindered by gender roles, although the specific impact has not been extensively investigated (42).

Overall, video remote interpreting offers great potential in many sectors, especially with respect to patient safety in health care. However, the enabling and hindering factors as seen from the perspective of interpreters with extensive experience in the medical VRI setting has not yet been subjected to scientific research. Exploring the perspectives of experienced professional medical interpreters in VRI is crucial for improving the quality of this service.

Therefore, this study aims to explore the factors that influence video remote interpreters’ job satisfaction and experience of stress, focusing on the role of work experience, supervision and gender, using quantitative methods. It is hypothesized that greater experience in interpreting generally and VRI specifically may be associated with lower levels of chronic stress and job dissatisfaction, as experienced interpreters may have developed more effective coping strategies over time. Additionally, the study aims to investigate whether supervision positively impacts interpreters’ stress levels and job satisfaction, while considering potential gender differences.

However, besides the above mentioned theoretical derived factors VRI settings might give rise to further important hindering and enabling factors for interpreters. To explore this broader research question, we added a qualitative study part. The objective of this analysis is to uncover these specific challenges and facilitators that interpreters encounter in the VRI setting in great detail, providing valuable insight into the complex factors that shape their work environment.

## 2 Materials and methods

This multi-methods study was reviewed by the Ethical Review Board for the Viennese Hospitals in the Vinzenz Holding (Study ID: I/2023). Because it was an online survey study, participants voluntarily participated, and no personal identifiable information was collected, it was deemed exempt. We adhered to the Declaration of Helsinki of 1975, as revised in 2000, and the EU GDPR throughout the study. Although no written consent was required from the participants, electronic informed consent was obtained prior to their participation in the survey.

### 2.1 Participants

Data was collected through an online questionnaire issued to interpreters working for *SAVD Videodolmetschen GmbH* (25), in their role as either employees or freelance interpreters. An invitation to participate in the survey including a link to the online questionnaire was sent to a total of 286 interpreters. Data was gathered between April 23 and June 23, 2020, with two reminders being sent out during this period. It should be noted that this period followed the first COVID-19-lockdown in Austria and Germany, during which regulations minimized physical contact, especially in vulnerable settings such as hospitals, prisons and public authorities.

A total of *N* = 87 persons completed the questionnaire in full, representing a response rate of 30.42%.

All participants had VRI experience as a result of working for *SAVD* and worked in the medical field, as well as legal and/or social fields. Most participants were female (67%, *n* = 58). The mean age of the respondents was 47.0 ± 12.8 years. The mean number of years spent working as an interpreter was 15.4 ± 11.9 years. Most respondents (77%, *n* = 67) worked as freelancers for *SAVD*, the rest were employees. The majority of interpreters (56%) had extensive experience in providing video/telephone interpretation services and had worked for *SAVD* for more than 3 years. The average estimate of the percentage distribution of perceived activity as a VRI in the medical sector (one of three areas of application: medical, legal and social services) was 44.23%, making it the most frequently requested field according to the responses of the interpreters surveyed. Participant characteristics are shown in Table 1.

**Table 1:** Participant characteristics [*mean (SD)* or *% (n)*].

Variable	*Mean (SD)* or *n (%)*	*N*
Sex, *n* (%)		87
Female	58 (66.7%)	
Age (in years), *M* (*SD*)	46.99 (12.85)	84
Working as an interpreter (in years), *M* (*SD*)	15.52 (11.95)	87
Experience as a video/tele remote interpreter prior to current position (yes/no), *n* (%)		87
Yes	19 (21.8%)	
Working experience as video remote interpreter total (%)		87
0–6 months	10 (11.4%)	
7–12 months	5 (5.7%)	
13–18 months	4 (4.6%)	
19–24 months	10 (11.4%)	
25–30 months	6 (6.9%)	
31–36 months	3 (3.4%)	
>36 months	49 (56.3%)	
Working as a telephone remote interpreter within current position (yes/no), *n* (%)		87
Yes	56 (64.4%)	
Experience in face-to-face interpreting (yes/no), *n* (%)		87
Yes	71 (81.6%)	
Working condition, *n* (%)		87
Freelance	67 (77%)	
Employed	20 (23%)	
Average time spent on interpreting assignments per week, *n* (%)		87
0–5 h	21 (24.1%)	
6–10 h	24 (27.6%)	
11–15 h	18 (21.8%)	
16–20 h	12 (13.8%)	
21–25 h	4 (4.6%)	
26–30 h	4 (4.6%)	
>30 h	3 (3.4%)	
Average duration of one interpreting assignment		87
0–15 min	7 (8%)	
16–30 min	59 (67.8%)	
31–45 min	21 (24.1%)	
>45 min	0 (0%)	
Number of average interpreting assignments per month for freelance interpreters, *n* (%)^1^		67
1–10	20 (29.9%)	
11–20	17 (25.4%)	
21–30	14 (20.9%)	
>30	16 (23.9%)	
Extent of employment (hours per week), *M* (*SD*)*, (Min-Max)*^2^	28.05 (10.00), (10–40)	20
Estimate of the percentage distribution of own activity as a VRI in the following three areas of application (in percent) *M* (*SD*)		83
Medical VRI	44.23 (17.39)	
Social services VRI	38.76 (14.25)	
Legal VRI	16.98 (9.48)	
Participation in supervision (yes/no), *n* (%)		87
Yes	24 (27.6%)	
Chronic stress score^3^, *M* (*SD*), *(Min-Max)*	20.74 (7.18) (12–45)	87
Job dissatisfaction score^4^, *M* (SD), (*Min-Max*)	13.90 (5.25) (8–30)	87

N/n, Number, M, Mean; SD, Standard Deviation.

1 Only participants working as freelancers for SAVD were asked this question.

2 Only participants working as employees for SAVD were asked this question.

3 Total Score of the standardized scale to assess chronic stress, (score could range from 12 = low to 60 = high stress).

4 Total Score of the standardized scale to assess job dissatisfaction, (score could range from 8 = low to 40 = high dissatisfaction).

### 2.2 Data collection

We included quantitative and qualitative questions in the online questionnaire, to gain a comprehensive overview of VRI experiences. It should be noted that interpreters employed by *SAVD* possess a diverse range of experience, not only in VRI, but also in telephone and on site interpretation. If applicable, these various modes of interpretation were incorporated into the survey alongside VRI to provide a comprehensive view of the interpreters’ expertise across different settings. We programmed the online questionnaire utilizing the website q-set.de.

#### 2.2.1 Quantitative data collection

A questionnaire set consisting of subscales of a standardized questionnaire (43), self-generated questions (related to professional activity, use of supervision, etc.), and socio-demographic questions (sex, age, etc.) was used.

The variables chronic stress level and job dissatisfaction were measured separately using the following two subscales of the German version of the standard questionnaire The Trier Inventory for Chronic Stress (*Trierer Inventar zum chronischen Stress: TICS*) which is recognized for its reliability and validity. Chronic stress was assessed using the 12-item screening subscale for chronic stress (SSCS), capturing the frequency of self-perceived stress within the last 3 months in five different domains of stress: work-related and social overload, chronic worrying, excessive demands, and lack of social recognition. Job dissatisfaction was measured using the 8-item TICS subscale (UNZU), which assesses stress resulting from a lack of need for job satisfaction (43). All items of the TICS scales were rated on a 5-point Likert scale (*0 = never, 1 = rarely, 2 = sometimes, 3 = often, 4 = very often*). Higher scores on both the chronic stress and job dissatisfaction subscales indicate greater levels of perceived stress and job dissatisfaction, respectively.

The participants also completed questions about their professional experience. Specifically, they provided information on how long they had worked in VRI and the volume of remote interpreting hours worked on behalf of *SAVD*. Participants further responded to questions about the percentage of their professional activity undertaken in specific domains, namely the social, medical, and legal areas.

To analyze whether supervision affects chronic stress or job dissatisfaction levels, the participants were asked if they had engaged with the voluntary supervision services, requiring responses in a binary format (yes/no).

The questionnaire included a further set of questions that were not intended for empirical study purposes. These included mainly a quality assessment to evaluate the supervision. These questions can be seen in the translated questionnaire included in the Supplementary material.

#### 2.2.2 Qualitative data collection

The qualitative section of the questionnaire comprised the following open-ended questions, designed to elicit comprehensive insights into the hindering and enabling factors as experienced by interpreters. The focus was on VRI as all interpreters in the survey had experience in VRI. In those interpreters who indicated having additional experience in telephone or in-person interpretations face-to-face, we also asked for the enabling and hindering factors for these interpretation modes. It should be considered that, due to the sequential structure of the survey, where questions regarding VRI settings were always presented first, responses related to telephone and in-person settings may have been influenced (i.e., participants might only incompletely state certain factors in later, as they have been previously mentioned). In light of the study’s main goals the focus of the analyzes was therefore placed on the answers from the VRI setting. The qualitative questions were as follows:

VRI Interpretation:

- When you think of your daily professional routine as a VIDEO remote interpreter what pressures, difficulties and challenges cross your mind?
- In your experience, what are the advantages of VIDEO remote interpreting?
  For those indicating having conducted telephone or face-to-face services:
- When you think of your daily professional routine as a TELEPHONE remote interpreter what pressures, difficulties and challenges cross your mind?
- In your experience, what are the advantages of TELEPHONE remote interpreting?
- When you think of your daily professional routine as a FACE-TO-FACE interpreter what pressures, difficulties and challenges cross your mind?
- In your experience, what are the advantages of FACE-TO-FACE interpreting?

### 2.3 Data analysis

The quantitative and qualitative data are reported in two sections: the first comprises the quantitative analyses, and the second focuses on the qualitative data.

#### 2.3.1 Analysis strategy for quantitative data

We analyzed whether work experience (interpretation experience in years, VRI experience in months) and share of a specific type of interpretation translation (law, medical, social service VRI, as a percentage) correlates with chronic stress and job dissatisfaction as measured on Trier Stress Inventory (TICS) scales. Due to the ordinal character of some of the variables, Spearman correlations were calculated (see Results 1). All analyses were conducted using the ggcorrplot package (0.1.4) in R (4.2.2).

To uncover, whether supervision has an effect on stress and job dissatisfaction we conducted group comparisons. In these comparison we considered gender as an additional factor as it has been proven to be influential on stress experience and job dissatisfaction. The two TICS subscales chronic stress and job dissatisfaction were separately analyzed in a 2 (supervision vs. no supervision) × 2 (gender: female vs. male) between subject factorial design (see Results 2). As the raw data sometimes showed extreme yet valid values for stress and dissatisfaction, (see also raw values graphically presented in section 3.1.2.), statistical test assumptions (e.g., sphericity) for classical ordinary least squares (OLS) were not met (determined by visual inspection and model assumption plots). Therefore, we opted for the robust regression model suggested by Yohai (44) based on the MM-estimator. Calculations were carried out using the robust::lmRob function and package. As we were mainly interested in group differences, the outcomes are reported as Analysis of Variance (ANOVA) tables using the Wald statistic (45).

#### 2.3.2 Analysis strategy for qualitative data

The qualitative analysis encompasses two open-ended questions addressing the challenges and advantages of video remote interpreting. Where available or feasible, additional questions about telephone remote and in-person interpreting were considered as a comparative analysis.

##### 2.3.2.1 Thematic content analysis

The answers to the questions were evaluated by three scientists using thematic content analysis (46, 47). They first familiarized themselves with the data by reading and rereading the interpreters’ responses, to gain a general overview and search for possible meanings and patterns. Initial codes and subcodes were then derived from the data in order to identify themes and establish a coding system with which to assign the interpreters’ individual responses or response sequences. When necessary, new codes were created, modified, or restructured during the process. Finally, the frequencies of the codes in the responses were quantified in order to identify key themes, and these are subsequently described and discussed. Identifying hindering and enabling factors specific to the VRI setting was achieved by coding responses to the questions on VRI, with consideration given to responses relating to the other two settings. If a respondent provided identical answers to questions about both remote interpreting (VRI and TRI) and in-person interpreting, it was assumed that this pertained to interpreting in general and was not specific to VRI. Consequently, these responses were not included in the analysis.

##### 2.3.2.2 Network analysis

In a further analysis step, we conducted a network analysis to visualize the qualitative outcomes of hindering and enabling factors in VRI (48). All respondents highlighted a range of factors; no respondent focused only on a single factor. To uncover the relational structure of these factors across all respondents we analyzed their frequency (How often are factors mentioned?) and co-occurrences (How often are factors mentioned together?). This helps to understand which factors are commonly expressed together and are considered relevant. The network analysis reveals numerous ambivalences and links between hindering and enabling factors, which are reported accordingly. We used the igraph and ggraph package in R for the network analysis (49, 50).

## 3 Results

### 3.1 Findings—quantitative analysis

#### 3.1.1 Results 1: correlations—what variables relate to chronic stress and job dissatisfaction?

**Figure 1:**
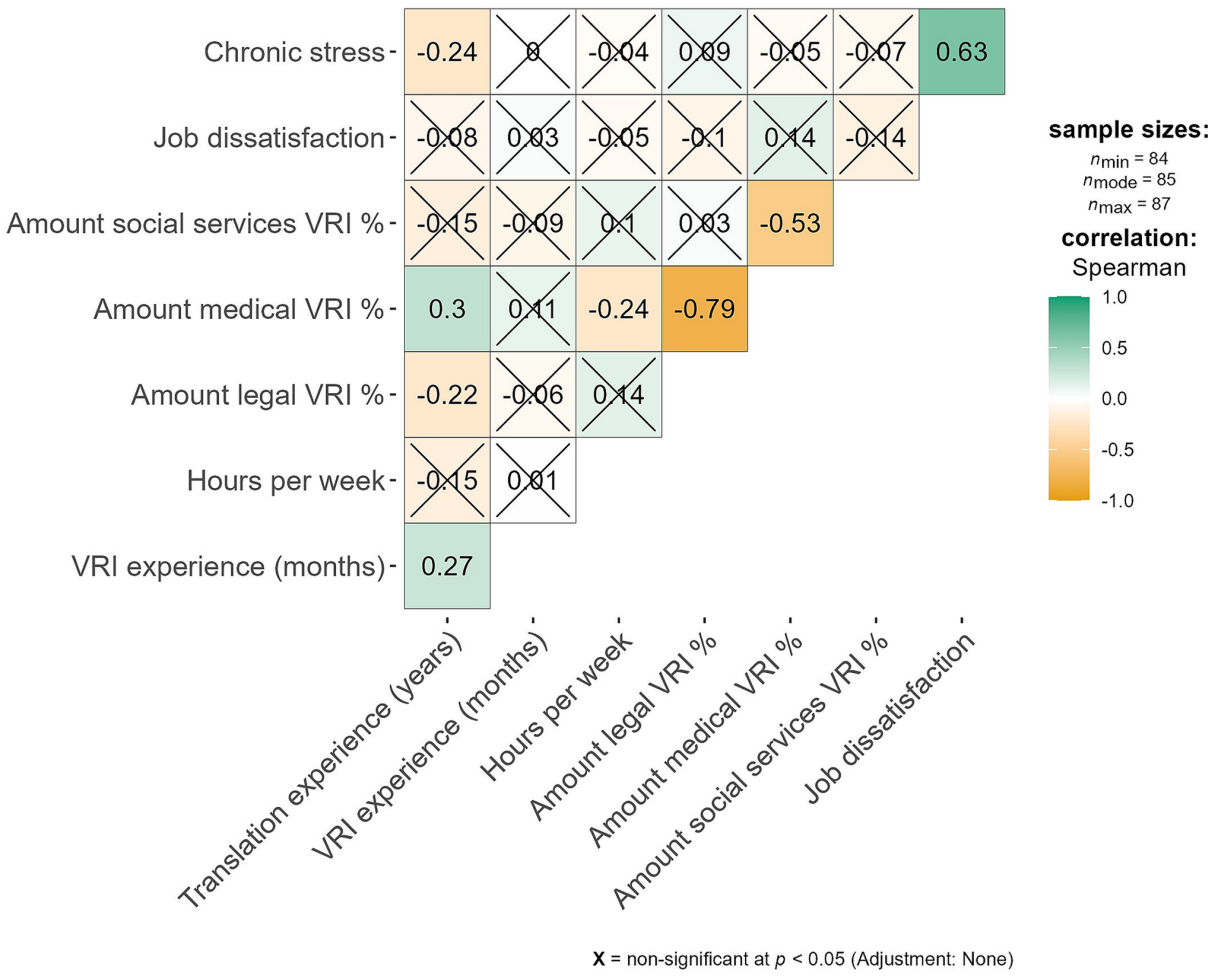
Correlations between work experience and interpretation type on chronic stress and job dissatisfaction.

Figure 1 shows the correlational results; non-significant correlations are flagged with a cross. Chronic stress and job dissatisfaction barely related to any of the above-mentioned variables. Only years of general interpretation experience were negatively related to chronic stress (rho = −0.24), with more experience linked to less chronic stress.

#### 3.1.2 Results 2: differences in chronic stress and dissatisfaction related to supervision and gender

**Figure 2:**
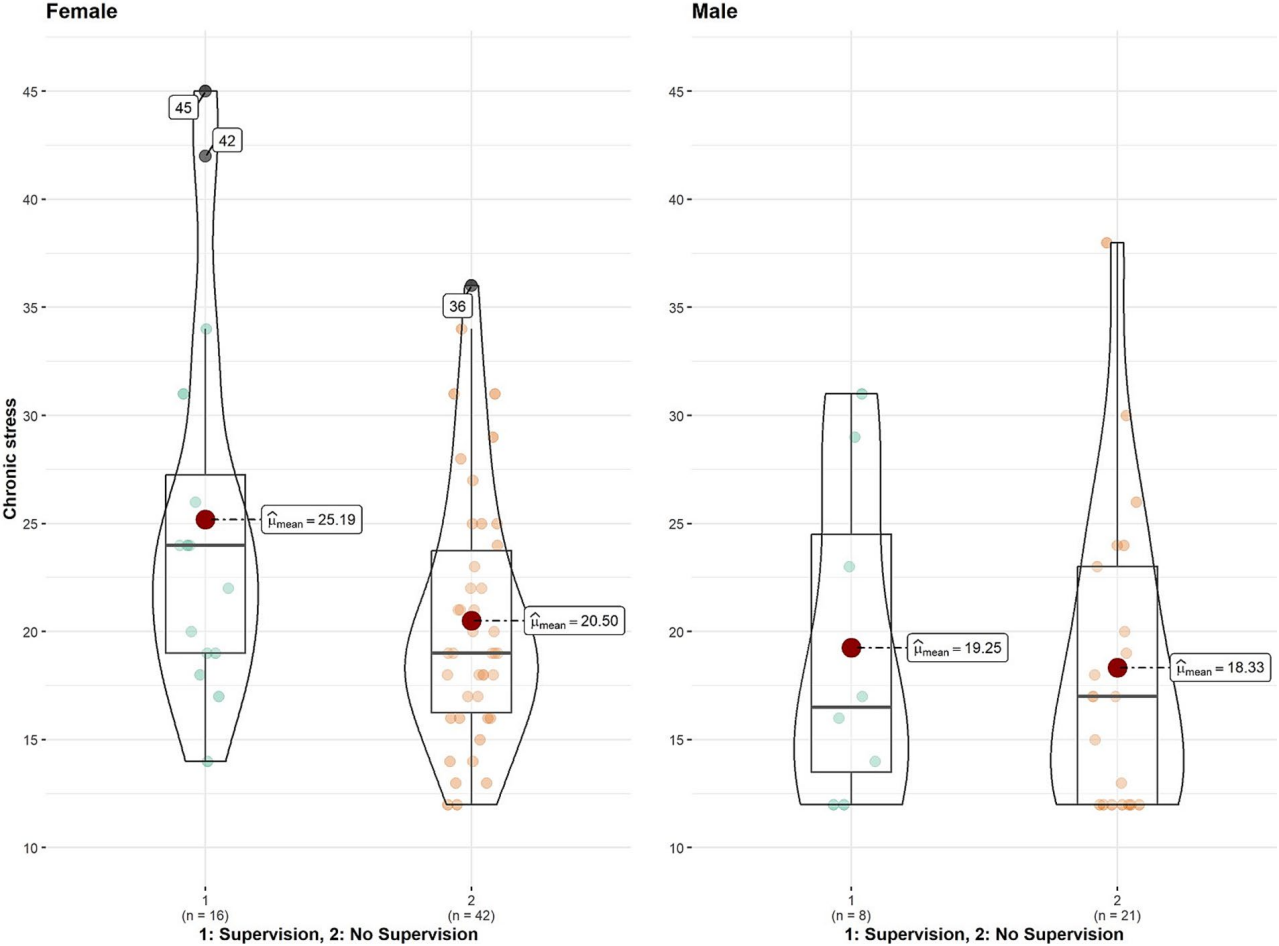
Chronic stress by Supervision × Gender.

**Figure 3:**
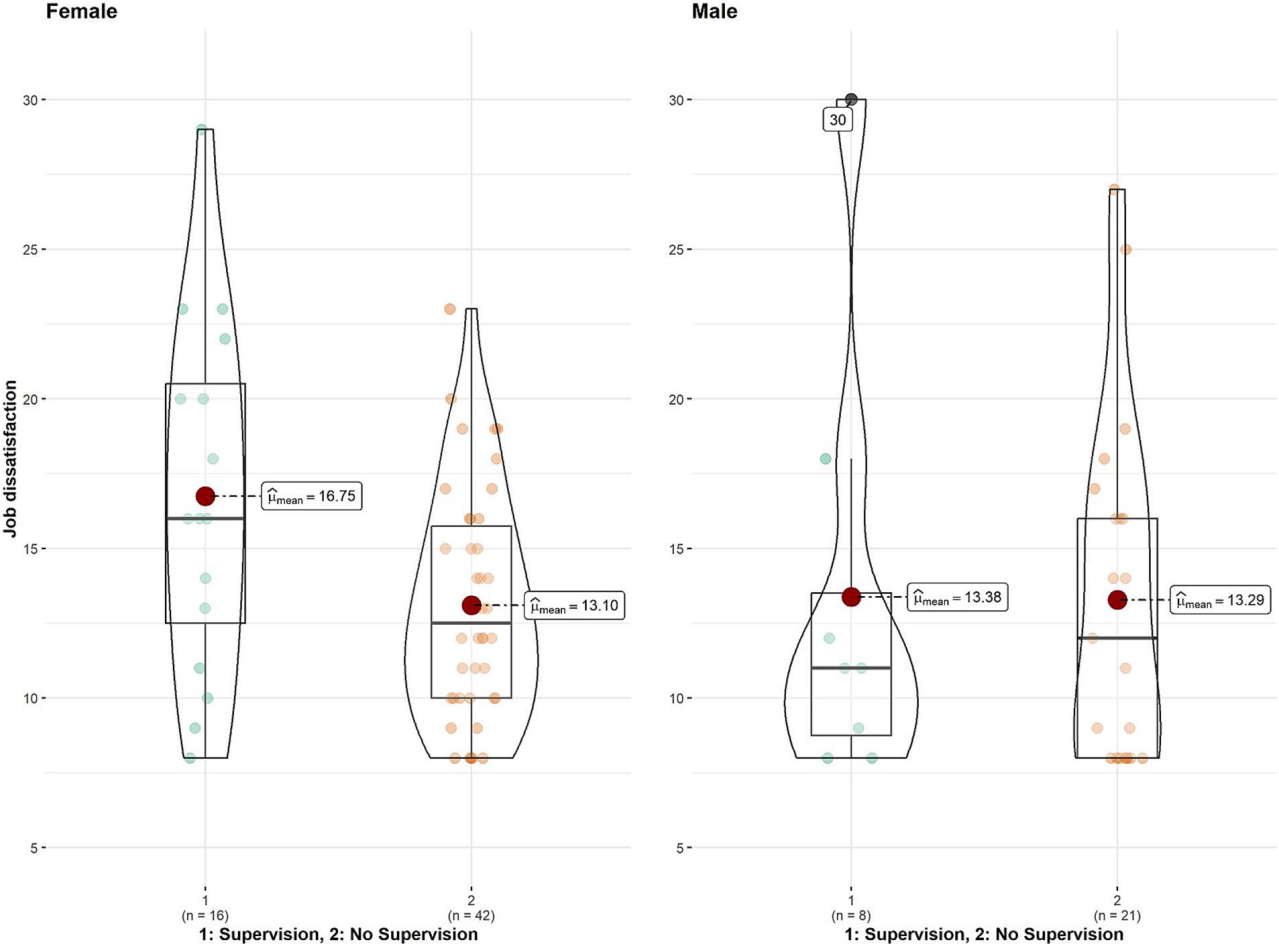
Job dissatisfaction by Supervision x Gender.

Analysis of the chronic stress levels did not yield any significant differences with regard to supervision. However, there was a trend of female interpreters reporting higher levels of chronic stress (see Table 2, and Figure 2, for statistics, raw data, dispersion and descriptives). With respect to job dissatisfaction, again supervision had no effect on reported dissatisfaction levels. Nonetheless, female interpreters reported significantly higher levels of dissatisfaction (see main effect of dissatisfaction, Table 3, and Figure 3).

**Table 2:** Chronic stress by Supervision × Gender.

	df	𝛘^2^	*p*(>Wald)
(Intercept)	1.000		
Supervision	1.000	1.508	0.220
Gender	1.000	3.033	0.082^†^
Supervision × Gender	1.000	0.019	0.891

*R2 = 0.062.*

^†^*p* < 0.1; * < 0.05; ** < 0.01; *** < 0.001.

**Table 3:** Job dissatisfaction by Supervision × Gender.

	df	𝛘^2^	*p*(>Wald)
(Intercept)	1.000		
Supervision	1.000	0.545	0.460
Gender	1.000	5.409	0.020*
Supervision × Gender	1.000	2.480	0.115

*R2 = 0.081.*

*p < 0.05; ** < 0.01; *** < 0.001.

### 3.2 Findings—qualitative analysis

#### 3.2.1 Thematic content analysis

Twenty-one factors were identified in the thematic content analysis, 14 factors were identified as hindering factors and seven factors as enabling factors.

The most frequently noted factors are described in more detail below, followed by the most important connections and ambivalences. Descriptions of all the codes are listed in Table 4.

**Table 4:** Qualitative analysis: list and description of codes.

Code (short)	Code (long)	Description
Hindering factors		
None	No hindering factors	The participant stated that there are no problems in the VRI setting
Technical	Technical problems	Technical problems were either mentioned in general terms or as specific technical problems, such as insufficient internet connection (on the customer side, e.g., in the hospital), sound or image quality, and sometimes also the difficulties this causes, such as gaps in understanding or disconnected calls.
No physical presence	Lack of physical presence	Difficulties mentioned arising from the lack of physical presence related either to spatial conditions (e.g., lack of visibility of all participants, lack of control over the setting), or “soft factors” (e.g., patient insecurity due to unfamiliar setting, interpreter is not sufficiently perceived as a human being).
Ad hoc situation	Difficulties due to the *ad hoc* nature of the situation	This code included, for example, a lack of opportunities to prepare for appointments due to a lack of advance information, a lack of introduction or necessary contextual information at the beginning of conversations, frequent switching between different fields of interpretation, etc.
Physical complaints	Physical complaints	The physical complaints described included problems caused by physical inactivity (e.g., tension) and VDU work (e.g., eye fatigue) in the VRI setting.
Non-verbal	Difficulties relating to facial expressions and gestures	This included both limited perception of the facial expressions and/or gestures of the conversation partners, and conversely the need for the interpreter to control their own facial expressions in the VRI setting (compared to telephone remote interpreting).
Work from home	Hindering aspects of the home office-environment	The difficulties noted with VRI in the home office setting were a lack of separation from emotional content, separation between work and private life, and ensuring the framework conditions for work on one’s own responsibility.
COVID-19 measures	Difficulties due to COVID-19 measures	Answers in this code describe acoustic comprehension difficulties due to safety measures such as wearing masks during the COVID-19 pandemic.
Emotional drain	Emotional drain	Emotional stress was generally cited as a challenge, while stressful topics (e.g., violence, illness, refugee experiences) and settings (e.g., psychiatry, palliative care) were also mentioned.
Linguistic difficulties	Difficulties in linguistic comprehension	In addition to linguistic difficulties in general, dialectal issues, communication barriers with non-native speakers of the interpreted language, unclear language, the use of technical language or abbreviations, and language-related challenges linked to mental health were explicitly highlighted.
Speaking discipline	Difficulties concerning communication	This code concerns special features of communication in VRI that are not considered by the conversation partners, such as several people speaking at the same time, no pauses in speech in which to translate what has been said, etc.
Role incompatibility	Interpreting as a mediator vs. maintaining impartiality	This code relates to difficulties in situations requiring the interpreter to mediate in the role of a language and cultural translator while at the same time maintaining impartiality (e.g., conflicts, cultural differences between the interlocutors and insufficient education/knowledge, lack of professionalism amongst healthcare or social staff, emotional situations).
Non-compliancy appointment	Difficulties because of scheduling problems	Where interpreting services are scheduled, this refers to lateness, non-appearance or untimely cancelation notice.
Gender	Gender of interpreter	The gender of the interpreter cannot be selected, which can lead to problems in specific situations and conditions.
Additional interpreter	Additional in-person interpreter	Clients or patients bring their own interpreter to the appointment.
Enabling factors		
None	No enabling factors	The participant stated that there are no enabling factors in the VRI setting.
Efficacy	Efficacy	Answers regarding efficacy included the elimination of travel and waiting time, the possibility of timely and cross-border interpreting support, flexibility, and the reduction of costs.
Safety	Feeling of safety	Within this code, the advantages of physical distance were mentioned generally, with regard to infectious diseases, or in potentially dangerous situations. In addition, anonymity and the protection of privacy as well as the possibility of regular earnings were described as advantages that provide a sense of security.
Varied content	Diversity of conversations to be interpreted	The participants described the variety of topics as making interpreting more diverse and interesting, as well as offering the opportunity to constantly learn new things, such as technical terms.
Emotional distance	Emotional detachment and neutrality through physical distance	In this context, the participants described how physical distance also enables them to better distance themselves psychologically and emotionally, i.e., in emotionally charged topics or conflict discussions. This facilitates a heightened focus on the core task of interpreting.
Work from home	Favorable aspects of the home office environment	This code pertains to favorable aspects of the home office environment, encompassing features such as a tranquil and relaxed ambiance, as well as a workspace customizable to individual preferences.
Auxiliary material	Auxiliary material	Video Remote Interpreting (VRI) provides the flexibility to utilize supplementary resources, including online dictionaries.
Visibility	Visibility due to video	Advantages over telephone Remote Interpreting (TRI) were highlighted, specifically noting that facial expressions and gestures are, in principle, visible in VRI.

##### 3.2.1.1 Hindering factors

###### 3.2.1.1.1 Technical difficulties

Technical problems in the VRI setting were the challenge most frequently mentioned and noted by almost 60% of the respondents. They were either mentioned in general terms or as specific technical problems, such as insufficient internet connections and lack of sound or image quality which make interpreting more demanding. Hospitals were mentioned several times in terms of technical problems, as this quote shows: “*Image quality, sound quality, positioning of the camera on the customer’s device, especially when used in hospitals.*”

Sometimes technical problems created additional difficulties, which are described in detail in the network analysis, see section 3.2.2.

###### 3.2.1.1.2 Lack of physical presence

A third of the interpreters described difficulties caused by the lack of physical presence. The spatial circumstances make the interpreting situation more difficult, especially in conversational constellations in which the interpreter has more than two dialogue partners. In some situations, not all participants in the conversation are visible via the video, and interpreters may be unaware of other, unseen, dialogue partners in the room until they suddenly enter the conversation. This lack of metacommunication makes interpretation significantly more difficult. It also creates a hierarchy of communication, in which contributions by the participant who is not physically present are more easily overlooked or overheard. This leads to uncertainty among the interpreters, but also among the clients, especially when unfamiliar with video services. As one respondent stated: “*For some clients, involving an interpreter via video conferencing is an unfamiliar experience, and they feel inhibited*.” The interpreter usually is also unable to view the location fully, or the specific spatial conditions. As a result, it is not possible to adequately assess the situation and all the participants. Therefore, the interpreter may feel a loss of control over the dialogue setting due to being connected remotely via a digital device rather than physically present. This also encourages interpreters to feel that they are sometimes not recognized or sufficiently “*perceived as a human being, but as a computer program.*” One respondent described the situation as follows: “*It is difficult to fully occupy one’s own place in a situation when you are not physically present.*” Overall, physical distance is described as making communication more difficult.

###### 3.2.1.1.3 Emotional drain

Emotional stress is also a topic frequently raised. It is noted and described by 22% of the respondents, both in general and specific settings or with respect to particular content. The conversations often involve stressful topics, such as physical and mental illness, the difficult psychosocial circumstances in which clients find themselves, traumatic experiences of violence or flight, or issues affecting children, whether illness or abuse. Furthermore, the settings in which the conversations are interpreted can also feel stressful, for example, in the palliative care setting, in psychological, psychotherapeutic or psychiatric conversations, in prisons, and in conversations with child and youth welfare services. In such situations, interruptions to conversations resulting from technical difficulties as described above are seen as particularly stressful, as they add an additional layer of stress to an already challenging situation.

VRI conducted by the interpreter at home is described as particularly distressing, as one interpreter points out: “*Interpreting at home via video gives you the feeling that you cannot quite separate yourself from the client. This is most stressful when interpreting for prisoners or people with serious mental illnesses.*”

###### 3.2.1.1.4 *Ad hoc* situation

Remote interpreting and the frequently *ad hoc* situation in which interpreters find themselves present them with several specific challenges not faced in in-person interpreting, as around 21% of the respondents reported. Often they are unable to prepare or plan for the appointments in advance because of a lack of necessary information: “*Interpreting without background knowledge or being told by the client what it is about.*” The interpreters report that the parties involved are often not introduced before or during the appointment; that background information is not provided and is therefore missing; and that they are often not told who has requested the interpreting service.

One answer clearly indicates what makes the interpreting situation hard for the interpreters: “*Every time there’s a call, you feel you are being thrown in at the deep end. Often you do not even know which city (…) the person is calling from, and you have little or no information about the context*.”

Often patients have previously spoken with other VRI interpreters. Consequently, the interpreter’s lack of knowledge of earlier conversations—about which the dialogue partners assume the interpreter is briefed—is an additional challenge.

As a result, meeting introductions are very abrupt, information is missing, and the meetings start and end quickly. The interpreters often join a dialogue which is already underway, requiring them to first familiarize themselves with the situation and causing them to miss any details previously discussed. Over the course of a series of individual assignments, interpreters need to switch quickly between the different areas of operation and “*the sudden swap from medicine to social affairs to law*” is often challenging. Interpreting services are required in a range of different fields (social services, medicine, law), giving interpreters hardly any possibility to reject certain topics and specialize in others. The survey participants reported having few breaks, and often needing to interpret unexpectedly long conversations, placing high demands on their ability to concentrate.

##### 3.2.1.2 Enabling factors

###### 3.2.1.2.1 Efficacy

However, VRI also has several advantages. The greatest benefit (noted by 61% of the interpreters surveyed) lies in the increased efficiency that comes with working remotely. This enables prompt and cost-efficient support which is flexible and allows ad-hoc translations. Travel and waiting times and costs are eliminated through the use of technical devices. I can cover a “*larger geographical area and therefore more orders, as I have no travel time*,” as one interpreter stated. In addition, interpreters can work across borders, irrespective of whether clients are based in Germany, Austria, or Switzerland.

###### 3.2.1.2.2 Work from home

The ability to work remotely from anywhere is another enabling factor, recognized by almost 38% of the respondents. As a rule, the respondents described having a standard home office setup, although it would theoretically be possible to work from anywhere offering a stable internet connection and a quiet environment. Respondents reported enjoying working in this more relaxed setting, especially as the workplace can be set up according to personal requirements and with all the necessary tools “*such as specialist dictionaries and electronic glossaries.*” Working from home is also perceived as being quiet and described as comfortable. One respondent even stated that working from home “*means I work in my favorite place. I save a lot of time because I do not have to drive to work or drive home afterwards. I really appreciate that.*”

###### 3.2.1.2.3 Varied content

Approximately 18% of the respondents highlighted the variety of the conversations they interpreted as a positive aspect, and as “*very enriching from a professional perspective.*” The diversity of the work and settings makes interpreting very varied, allowing interpreters to constantly expand their vocabulary and expertise, and to broaden their horizons.

###### 3.2.1.2.4 Safety

Remote interpreting offers interpreters safety on various levels, a factor highlighted by nearly 14% of the participants. The timing of the survey, at the height of the COVID-19 pandemic, must also be considered, with some respondents noting that VRI removed any risk of infection. Another health aspect is that remote interpreting removes the need for interpreters to enter dangerous environments such as prisons, or be around potentially aggressive patients in hospitals, thereby ensuring their physical safety. The anonymity—both for interpreters and clients—also contributes to the feeling of security, as well as neutrality, and the ability to maintain boundaries. As it is not possible to hold conversations before or after the appointment, any potential conflicts of interest that might occur during on-site appointments, for example, when clients and interpreters meet in the waiting room or when leaving the building, are also avoided. The video service also supports interpreters’ financial security, by allowing them to work even when many workplaces could not be entered or where access was severely restricted due to protective measures to prevent the spread of COVID-19.

###### 3.2.1.2.5 (Emotional) distance

Almost 14% of the participants repeatedly noted the physical distance created by VRI as allowing them to better distance themselves psychologically and emotionally when dealing with emotionally stressful topics or conflict discussions. This, in turn, enables them to focus more strongly on the core task of interpreting. One respondent described a feeling of greater detachment but with a simultaneous feeling of contact and involvement: “*There is more of a feeling of real contact/involvement than with telephone interpreting … It’s easier to distance yourself from the situation because you are not physically there*.”

#### 3.2.2 Network analysis: connections and ambivalences

**Figure 4:**
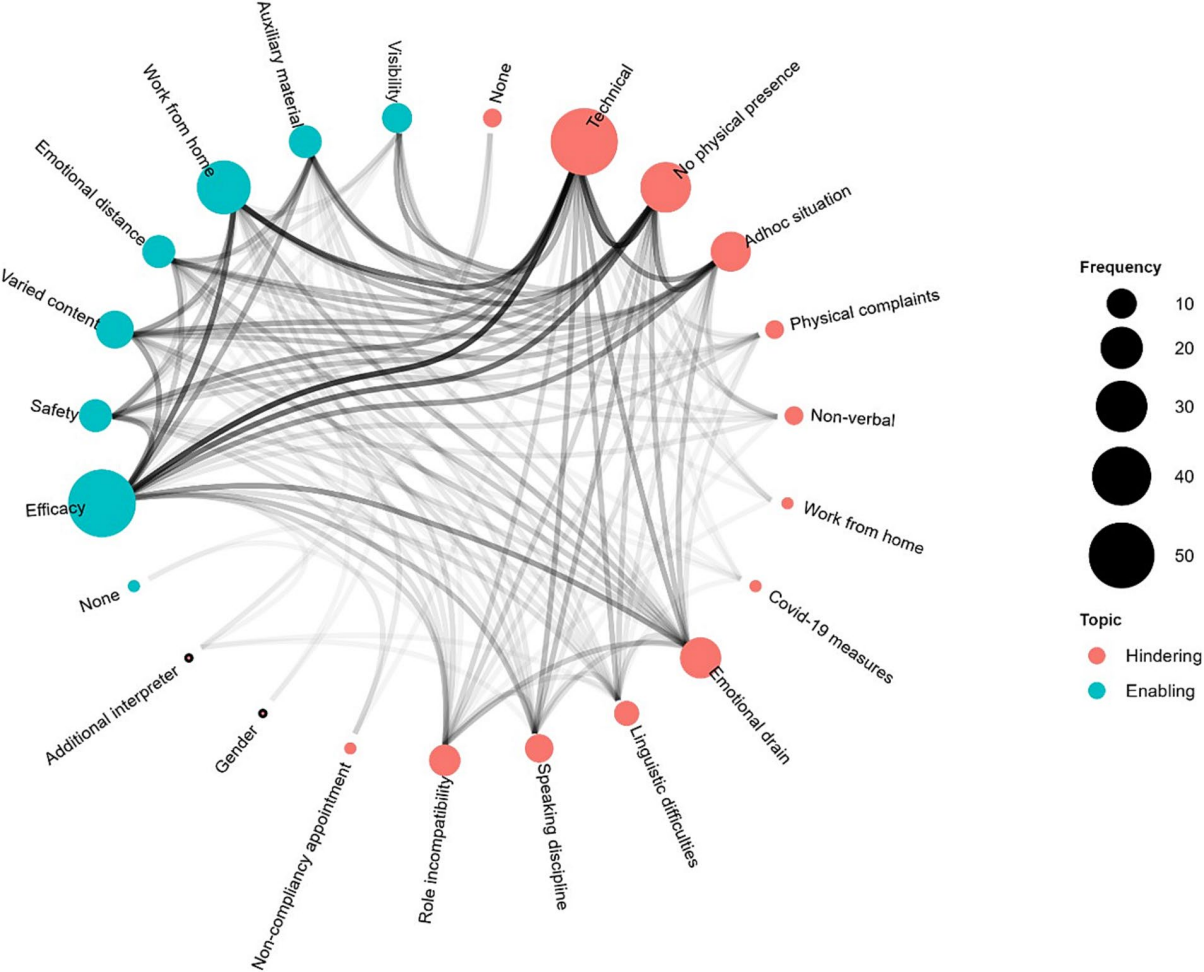
Enabling and hindering factors for VRI (hierarchical network graph). No participant mentioned only 1 factor. Factor type (hindering, red; enabling, blue), frequency of factor (size of bubble), and co-occurrence (darker lines represent higher co-occurrences) of named factors in the answers.

Figure 4 visualizes the frequency of occurrence and shows the links in the network analysis. Fourteen hindering factors (red bubbles) and seven enabling factors (blue bubbles) were identified in the VRI setting. The size of each bubble in the figure indicates how often the hindering or enabling factor was mentioned—the more often a factor was mentioned, the larger the bubble. The co-occurrence of factors amongst participants is shown by the connecting lines; the darker the line, the more often participants mentioned both factors.

Especially technical problems seem to create additional difficulties, with interpreters also describing gaps in understanding or disconnected calls. For example, one respondent stated that: “*Continuing a conversation that was cancelled due to technical problems*” was challenging because, as another respondent pointed out: “*As the second interpreter, [in such situations] it is difficult to familiarize yourself with the topic*.”

Another statement gives a particularly accurate description of the consequences of technical problems: “*Internet problems or acoustic interruptions or noise lead to gaps in understanding. This also makes it hard for the interpreter to question what has been said, and simply prolongs the conversation. This is especially stressful where the client becomes impatient and emotional as a result of the interruptions*.” Problems also arise when a particularly difficult conversation is unexpectedly interrupted: “*When a conversation is broken off in a mentally stressful situation, it is more difficult to distance yourself.*” Further, the ambivalences associated with remote interpreting become evident with the network analysis. As the following quote shows, the interpreters are aware that the advantages and disadvantages differ according to the various groups involved: “*I do NOT find video interpreting per se advantageous for the interpreters. However, this form of interpreting certainly makes sense in some settings and is beneficial for the other participants, partly because it keeps the costs very low*.” On one hand, the interpreters recognize the problems caused by technical difficulties when conversations cannot be sufficiently well followed or break off abruptly, the lack of physical presence and the atmospheric aspects that are lost as a result, as well as the disadvantages that the *ad hoc* situation brings with it when interpreting remotely. Yet at the same time, the respondents appreciate the efficiency that comes with working remotely. There is no need to travel and the time between individual assignments is minimized.

There is a similar connection between the hindering factors of technical difficulties, lack of physical presence, and the disadvantages of ad hoc situations, and the advantages offered by working from home in a relaxed and quiet environment, with a workplace designed according to the interpreter’s specific needs. One respondent commented on the positive and negative aspects of working remotely: “*I cannot prepare myself for the calls. With the new program, I often do not know who is calling.*” And then he or she points out: “*I do not need to travel and still have pretty good contact with customers*.”

With respect to emotional stress in the VRI setting, analysis of the interpreters’ responses highlights differences in the study sample: While some interpreters cited psychological burden as a hindrance in both remote and in-person interpreting settings (suggesting a lack of discernible differences in this burden according to setting), alternative perspectives may highlight nuances or distinct challenges in each environment. However, where an interpreter gave the same statement for more than one setting, the code for frequency and network analysis was not counted. Some participants mentioned feeling emotionally drained only in the VRI setting, with others citing remote interpreting as an advantage in this respect, with the physical distance also allowing them to distance themselves better psychologically from (emotional) situations. These respondents generally also described emotional stress in the setting of in-person interpreting. However, some interpreters were ambivalent about emotional stress versus emotional distance. For example, when asked about hindering factors in the VRI setting, one interpreter stated that remote interpreting from home is stressful where the client is based in settings such as a prison or is suffering from severe mental illness. Yet the same respondent noted better emotional distancing as an enabling factor where the interpreter is not conducting VRI from their own home, but, for example, from an office space.

Working from home or on site are mutually exclusive scenarios. Although lack of physical presence is noted as a hindering factor by some interpreters, they also enjoy the benefits of working from home. However, working from home also depends on personal preferences, individual circumstances, and the topics being translated. Provided the conditions are right, i.e., a stable internet connection and a quiet working environment, the option of working from home is seen as positive. However, when particularly difficult cases are discussed, the lack of physical separation from home also makes it more difficult to set emotional boundaries. With respect to emotional factors, one respondent reported: “*When a conversation is broken off in a stressful situation, it is more difficult to mentally distance yourself*.” The changeover from work to private life is very abrupt, with no commute, for example, offering time for a smoother transition.

We also see a connection between the tendency for the *ad hoc* nature of video interpreting situations to act as a hindering factor, and the enabling aspects offered by the diverse range of topics with which interpreters deal. They appreciate the opportunity to constantly learn and tackle challenges that add variety to their working day, as two quotes from one respondent indicate: “*The variety of topics is a linguistic challenge*,” but “*You always learn something new, especially in medical and legal fields.*” Nevertheless, interpreters feel they could better adapt to the interpreting situation and perform their services to an even higher standard if given slightly more time to prepare or provided with advance information.

## 4 Discussion

This study investigated factors influencing the job satisfaction and stress levels of video remote interpreters, focusing on work experience, supervision, and gender. Additionally, it sought to gain a deeper understanding of the enabling and hindering factors specific to the VRI setting through qualitative analysis. Quantitative findings revealed that greater general interpreting experience was associated with lower chronic stress levels, though no significant differences were found concerning supervision attendance. However, significant gender differences emerged, with female interpreters reporting higher job dissatisfaction than their male counterparts. Qualitative analysis provided rich insights into the complex interplay of enabling factors and hindering factors. While VRI has been shown to increase efficiency and emotional distancing, and interpreters have expressed appreciation for the opportunity to work remotely and the diversity of the conversations they are interpreting, it has also been identified as a source of challenges. These challenges have been found to include, in particular, technical problems, difficulties arising from the lack of physical presence and emotional distress. These findings are further discussed below, along with practical recommendations to address identified challenges and improve working conditions for VRI interpreters.

According to our qualitative analysis of the interpreters’ responses, empathy appears to be a key factor in the VRI situation. The interpreters surveyed sometimes feel they are treated as a machine, due to the physical distance. Interpreters often deal with topics which are emotionally stressful for the clients. In the generally impersonal video setting, interpreters therefore often have a second function in addition to overcoming the language barrier, namely that of conveying empathy. Furthermore, in *ad hoc* situations, the interpreters sometimes must go beyond pure language mediation, needing to explain not only the language, but sometimes also the customs or legal situation of the foreign country. When asked what stresses, difficulties and challenges come to mind when thinking about everyday working life as a video interpreter, one interpreter replied that: *“You have to make the customer aware of the local understanding of the problems.”* Consequently, cultural differences also must be balanced out. Another interpreter says that: *“In the case of different systems, it is necessary to explain the situation to both parties.”*

For VRI to work satisfactorily for all parties, all sides need to understand the specifics of the situation. A further aspect identified by our qualitative analysis is the importance of respecting the needs of the dialogue participants, e.g., to allow sufficient time for translation, to avoid technical language and internal terms as much as possible, and to ensure a quiet environment as well as adequate technical requirements (e.g., stable internet connection). While some of these requirements are important in all interpreting situations, a quiet environment and the technical necessities are particularly important in VRI. The rustling of paper and several people speaking simultaneously severely reduces the sound quality and impairs the work of the interpreter and was repeatedly mentioned as a hindering factor by the respondents in our study. The importance of well-considered implementation as well as technical and spatial requirements has already been mentioned by interpreters in previous studies (20, 51, 52).

In conversation constellations involving many people, it is important to consider whether simple VRI is always the best solution. For example, a respondent says: “*When it comes to conversations in larger groups (…) a single fixed microphone is not sufficient for everyone present.*” Such situations therefore require prerequisites, such as several microphones or a mobile microphone of high quality, so that everything said can be understood and translated by the interpreters. As far as the camera setting is concerned, all the participants in the conversation need to be in the frame and therefore visible, i.e., special requirements are also needed here.

VRI enables interpreters to be called in quickly without having to travel as several respondents have noted in the open-ended questions, and is therefore ideal for *ad hoc* situations. However, as our qualitative analysis indicates, this is a challenge for the interpreter, as they can be called upon to translate unexpectedly, even in difficult conversations and situations, without any warning or lead time. It is also possible to make advance appointments for interpreting services, as is usual for face-to-face interpreting on site. A short lead time is useful and helps interpreters to prepare accordingly. One respondent says in this regard: “*Often long or difficult conversations are spontaneous, but where it would have been possible for the customer to make an appointment in advance, even if the lead time was just a few minutes.*” It is therefore clear that even little preparation is better than none. It would also be important to obtain precise information about the topic of the conversation so that the interpreters can prepare accordingly. This finding is consistent with a recent study (53): Apart from feeling guilty about delivering bad news, the most frequently noted concern among healthcare interpreters was not knowing the content prior to a discussion. Correspondingly, one of the most common suggestions for improvement was an advance briefing with medical teams prior to the interpreting situation. This concern would seem even more valid and/or relevant in the VRI context as our qualitative analysis has shown. Due to the efficient but at the same time fast-paced nature of remote interpreting, briefing seems to be neglected even more often in the VRI context than in conversations with in-person interpreters. Therefore, it is imperative to raise awareness among all VRI users of the importance of sufficient briefing in medical interviews, especially in the healthcare field.

In comparison to a previous study, which showed that the satisfaction of healthcare professionals increased the longer they worked with VRI (38), our analyses showed no substantive correlation between the duration of professional activity as a video remote interpreter and stress perception or job satisfaction. This suggests that interpreters who are new to the specific VRI setting do not necessarily experience more stress or (dis)satisfaction than their more experienced colleagues who have greater familiarity with the VRI setting. Nevertheless, correlation analyses indicated that greater general experience in the interpreting profession was associated with lower stress levels. This could indicate that interpreters who have more interpreting experience in general are better able to adapt to a different setting, namely the VRI. This can serve as an advantage in implementing VRI solutions. However, as outlined above, individual (stress) factors should be considered.

Generally, in our study, women reported higher levels of job dissatisfaction and (as a trend) more work-related stress than their male colleagues, similar to findings in other work environments (41). This could be due to gender roles, with women often having to deal with multiple stress factors, e.g., coordinating family and work (54). Another factor could be the interplay between the levels of stress and job dissatisfaction with supervision. Theoretically, engaging in supervision could reduce stress levels and dissatisfaction (39). On the other hand, greater stress and dissatisfaction might encourage interpreters to seek supervision, especially as interpreters participate voluntarily. We did not find clear outcomes with respect to these predictions. Descriptively, it appears that the group attending supervision reported higher levels of stress and job dissatisfaction, and this was additionally moderated by gender.

According to our qualitative analysis of the interpreters’ responses, there is still some level of uncertainty among clients and patients when it comes to using VRI. However, this may be because VRI is unfamiliar to this group. It is to be expected that once the service has been used a few times and become established, this will no longer be the case, also considering that in general there seems to be no difference in patient satisfaction between VRI and in person interpreting (19).

## 5 Limitations

It should be noted that the interpreters in the survey were recruited via a single company. Therefore, the results may not be directly or completely transferable to VRI services from other providers. However, due to the large number of freelance interpreters offering VRI on behalf of the company, it can be assumed that most respondents have a wealth of experience in a variety of settings. At the same time, the survey of stress levels and job satisfaction relates to the current professional context of remote interpreting, and with no comparison group of in-person interpreters. Future studies should therefore investigate the stress levels and job satisfaction of remote versus in-person interpreters.

It should be further noted that the survey of interpreters was conducted during the lockdowns in Austria and Germany and this may have impacted the results relating to stress and job dissatisfaction. While crisis periods and specifically the COVID-19 pandemic can lead to increased stress, the interpreters may have had a slightly lower workload during the lockdowns, and this may have also reduced their work-related stress levels in these periods.

## 6 Conclusion

Overall, this study indicates that video interpreting is perceived as an efficient and satisfactory alternative to interpreting on site. The interpreters surveyed described several enabling factors of VRI to a considerable extent alongside the obstacles encountered. Many of these hindering factors could be solved if recommendations were adhered to, but these must first be known, communicated, and implemented. Briefing and subsequently debriefing interpreters is recommended as a means of managing difficult conversations and reducing interpreter stress (53). As our study showed this is even more relevant in the VRI setting where, due to the *ad hoc* nature of the situation and time pressures, discussions before and following the interpreting sessions are often lacking. Another important recommendation is to ensure optimal technical and spatial conditions in the VRI setting. Satisfactory use of the interpreting services is important to all parties involved. Patients, physicians and interpreters all need to be on board for VRI to be successful. These insights could contribute to optimizing future VRI conditions, in turn improving outcomes for all parties involved and consequently also enhancing patient safety in healthcare settings involving linguistically diverse patient populations.

## Data Availability

The datasets presented in this article are not readily available because the data are not publicly available for ethical and privacy reasons. Requests to access the datasets should be directed to sophie.klomfar@lbg.ac.at.
