# Supplementary material for: Working experiences of remote interpreters in health care settings—insights from Austria and Germany
Source: Front Public Health. 2025 Feb 10;13:1477965. doi: 10.3389/fpubh.2025.1477965 (PMC11848680; doi:10.3389/fpubh.2025.1477965)
Supplement: Supplementary file 1 [file Data_Sheet_1.pdf]

Supplementary Material

1. Questionnaire

The questionnaire consisted of standardized questionnaires and a self -designed part: Two subscales (Short Screening Scale for Chronic Stress and work discontent/job dissatisfaction) from the questionnaire The Trier Inventory for Chronic Stress (*Trierer Inventar zum chronischen Stress: TICS*; (38) to quantify chronic stress and job dissatisfaction were included.

The self- designed questions were as follows, see below: Original German version and translation of items to English [in brackets].

Seit wie vielen Jahren sind Sie als Dolmetscher:in insgesamt tätig? [For how many years have you been working as an interpreter in total?]

___ Jahre [years]

Hatten Sie vor Ihrer Tätigkeit bei SAVD schon Erfahrungen mit Videodolmetschen /Telefondolmetschen? [Did you have any experience with video or telephone remote interpreting before working for SAVD?]

Ja [Yes]

Nein [No]

Wie lange sind Sie bei SAVD-Videodolmetschen tätig? [How long have you been working for SAVD video remote interpreting?]

0 - 6 Monate [0 - 6 months]

7 - 12 Monate [7 - 12 months]

13 - 18 Monate [13 - 18 months]

19 - 24 Monate [19 - 24 months]

25 - 30 Monate [25 - 30 months]

31 - 36 Monate [31 - 36 months]

über 3 Jahre [more than 3 years]

Welche Sprachen bieten Sie im Rahmen Ihrer Tätigkeit bei SAVD an? (Mehrfachnennungen sind möglich) [Which languages do you offer as part of your work at SAVD? (multiple answers are possible)]*

Albanisch [Albanian]	Amharisch [Amharic]	Arabisch [Arabic]	Armenisch [Armenian]	Aserbaidschanisch [Azerbaijani]
Bengalisch [Bengali]	Berberisch [Berber]	Bosnisch/Kroatisch/ Serbisch [Bosnian/ Croatian/ Serbian]	Bulgarisch [Bulgarian]	Chinesisch [Chinese]
Dari [Dari]	Englisch [English]	Farsi [Farsi]	Filipino (Tagalog) [Filipino (Tagalog)]	Französisch [French]
Fula [Fula]	Gebärdensprache (DGS) [German Sign language]	Georgisch [Georgian]	Griechisch [Greek]	Hausa [Hausa]
Hebräisch [Hebrew]	Hindi [Hindi]	Igbo [Igbo]	Italienisch [Italian]	Kurdisch [Kurdish]
Lettisch [Latvian]	Litauisch [Lithuanian]	Malinke [Malinke]	Mandinka [Mandinka]	Mazedonisch [Macedonian]
Mongolisch [Mongolian]	Nepalesisch [Nepalese]	Oromo [Oromo]	Paschtu [Pashto]	Polnisch [Polish]
Portugiesisch [Portuguese]	Punjabi [Punjabi]	Rumänisch [Romanian]	Russisch [Russian]	Slowakisch [Slovakian]
Slowenisch [Slovenian]	Somali [Somali]	Soninke [Soninke]	Spanisch [Spanish]	Suaheli [Swahili]
Susu (Mandesprache) [Susu (Mande language)]	Tamilisch [Tamil]	Thailändisch [Thai]	Tigre [Tigre]	Tigrinya [Tigrinya]
Tschechisch [Czech]	Tschetschenisch [Chechen]	Türkisch [Turkish]	Twi [Twi]	Ukrainisch [Ukrainian]
Ungarisch [Hungarian]	Urdu [Urdu]	Vietnamesisch [Vietnamese]	Wolof [Wolof]	Zarma [Zarma]

* Analyzes to these question that were not relevant to the research questions can be requested from the authors if needed.

Falls Sie mehrere Sprachen im Rahmen Ihrer Tätigkeit bei SAVD anbieten: Welche Sprache wird am häufigsten angefordert? [If you offer several languages as part of your work at SAVD: Which language is most frequently requested?]*

____

In welchem Arbeitsverhältnis sind Sie für SAVD Videodolmetschen tätig? [What is your working relationship with SAVD?]

Angestellt [employed]
Freiberuflich [freelance]

Wie viele Stunden pro Woche sind Sie bei der SAVD angestellt? [How many hours per week are you employed by SAVD?]

_______ h/Woche [h/week]

Wie viele Dolmetschungen führen Sie im Auftrag der SAVD im Schnitt pro Monat durch? [How many interpreting assignments do you carry out on behalf of the SAVD per month on average?]

1 - 10

11 -20

21 - 30

mehr als 30 [more than 30]

Wie viele Stunden pro Woche nehmen die Dolmetscheinsätze im Rahmen Ihrer Tätigkeit bei der SAVD durchschnittlich in Anspruch? [How many hours per week on average do you spend on interpreting assignments as part of your work at the SAVD?]

0 - 5 Stunden [0 - 5 hours]
6 - 10 Stunden [6 - 10 hours]
11 - 15 Stunden [11 -15 hours]
16 - 20 Stunden [16 - 20 hours]
21 - 25 Stunden [21 - 25 hours]
26 - 30 Stunden [26 - 30 hours]
mehr als 30 Stunden [more than 30 hours]

Wie lange schätzen Sie die durchschnittliche Dauer eines Dolmetschgesprächs im Rahmen Ihrer Tätigkeit ein? [How long do you estimate the average duration of an interpreting meeting as part of your work?]

0 - 15 Minuten [0 - 15 minutes]
16 - 30 Minuten [16 - 30 minutes]
31 - 45 Minuten [31 - 45 minutes]
46 - 60 Minten [46 - 60 minutes]

61 - 75 Minuten [61 - 75 minutes]
76 - 90 Minuten [76 - 90 minutes]
mehr als 90 Minuten [more than 90 minutes]

In welchen Einsatzbereichen sind Sie als VideodolmetscherIn bei SAVD tätig?
Bitte versuchen Sie eine prozentuale Aufteilung auf folgende drei Bereiche und geben Sie die Prozentzahl in Zahlen hier ein ("Medizin/Gesundheitsberufe" + "Recht" + "Soziales" = 100%): [In which areas do you work as a video remote interpreter at SAVD?
Please try a distribution on a percentage basis into the following three areas and enter the percentage as digits here ("Medicine/Healthcare professions" + "Law" + "Social affairs" = 100%):]

Medizin/Gesundheitsberufe [Medicine/Healthcare professions]: __________

Recht [Law]: __________

Soziales [Social affairs] : __________

Sind Sie bei SAVD auch als TelefondolmetscherIn tätig? [Do you also work as a telephone remote interpreter at SAVD?]

Ja [Yes]

Nein [No]

Sind oder waren Sie vor bzw. neben Ihrer Tätigkeit bei SAVD als Dolmetscherin VOR ORT tätig? [Before or in addition to your work at SAVD, did you work as an interpreter ON SITE?]

Ja [Yes]

Nein [No]

Wenn Sie an Ihren beruflichen Alltag als VIDEO-DolmetscherIn denken, welche Belastungen, Schwierigkeiten und Herausforderungen kommen Ihnen in den Sinn? Nennen Sie im Folgenden Belastungen, Schwierigkeiten und Herausforderungen im Setting VIDEO-Dolmetschen [When you think of your daily professional routine as a VIDEO REMOTE interpreter what pressures, difficulties and challenges cross your mind? List the pressures, difficulties and challenges in VIDEO REMOTE interpreting]

Wenn Sie an Ihren beruflichen Alltag als TELEFON-DolmetscherIn denken, welche Belastungen, Schwierigkeiten und Herausforderungen kommen Ihnen in den Sinn? Nennen Sie im Folgenden Belastungen, Schwierigkeiten und Herausforderungen im Setting TELEFON-

* Analyzes to these question that were not relevant to the research questions can be requested from the authors if needed.

Dolmetschen [When you think of your daily professional routine as a TELEPHONE REMOTE interpreter what pressures, difficulties and challenges cross your mind? List the pressures, difficulties and challenges in TELEPHONE REMOTE interpreting]

Wenn Sie an Ihren beruflichen Alltag als DolmetscherIn VOR ORT denken, welche Belastungen, Schwierigkeiten und Herausforderungen kommen Ihnen in den Sinn? Nennen Sie im Folgenden Belastungen, Schwierigkeiten und Herausforderungen im Setting Dolmetschen VOR ORT [When you think of your daily professional routine as a FACE-TO-FACE interpreter what pressures, difficulties and challenges cross your mind? List the pressures, difficulties and challenges in FACE-TO-FACE interpreting]

Welche Vorteile erleben Sie im Zuge des VIDEOdolmetschens? [In your experience, what are the advantages of VIDEO REMOTE interpreting?]

Welche Vorteile erleben Sie im Zuge des TELEFONdolmetschens? [In your experience, what are the advantages of TELEPHONE REMOTE interpreting?]

Welche Vorteile erleben Sie im Zuge des Dolmetschens VOR ORT? [In your experience, what are the advantages of FACE-TO-FACE interpreting?]

Was wäre Ihnen in der Zukunft zur Qualitätssicherung Ihrer Tätigkeit als VideodolmetscherIn wichtig? Sollte Ihnen eine nicht genannte Maßnahme einfallen, können Sie diese beim letzten Punkt nennen und dessen Wichtigkeit beurteilen. [What would be important to you to ensure the quality of your work as a video interpreter in the future? If you can think of a measure that is not mentioned, you can add it to the last field and rate its importance.]*

	1=gar nicht wichtig [not important at all]	2	3	4	5=sehr wichtig [very important]
Aus-/Fortbildung [education/training]					
Vorbereitung [preparation]					
Austausch mit KollegInnen [communication with colleagues]					
Supervision [supervision]					
Sonstige, nicht genannte Maßnahme [other measures not listed above]: ________					

Haben Sie das psychologische Supervisionsangebot bereits in Anspruch genommen? [Have you already made use of the psychological supervision service?]

Ja [Yes]

Nein [No]

Welche Angebote haben Sie bereits in Anspruch genommen? (Mehrfachnennungen sind möglich) [What services have you already made use of? (multiple answers are possible)]*

* Analyzes to these question that were not relevant to the research questions can be requested from the authors if needed.

Gruppensupervision [group supervision]

Einzelsupervision [individual supervision]

Präferieren Sie die Supervision im Einzel- oder im Gruppensetting? [Do you prefer supervision in an individual or a group setting]*

Einzelsetting [individual setting]

Gruppensetting [group setting]

Beide [both]

Keines von beiden [neither of them]

Wie häufig haben Sie insgesamt bereits an der angebotenen psychologischen Supervision (Einzel- und/oder Gruppentermine) teilgenommen? [How often have you participated in the psychological supervision offered (individual or group appointments)?]*

Einmal [once]

2 - 5 Mal [2 - 5 times]

6 - 10 Mal [6 - 10 times]

11 - 15 Mal [11 - 15 times]

mehr als 15 Mal [more than 15 times]

Würden Sie das von der SAVD zur Verfügung gestelltes psychologisches Supervisionsangebot (weiterhin) in Anspruch nehmen? [Would you (continue to) made use of the psychological supervision service provided by the SAVD]*

Nein [No]

Eher nein [Rather no]

Vielleicht [Perhaps]

Eher ja [Rather yes]

Ja [Yes]

Falls Sie das Angebot der Supervision nicht oder eher nicht in Anspruch nehmen möchten: Aus welchem Grund möchten Sie dieses Angebot nicht oder eher nicht in Anspruch nehmen? [If you (rather) do not wish to use the supervision service, what is the reason for not wanting to take advantage of this offer?]*

Wie sehr treffen die folgenden Aussagen bezüglich der Bedeutung der Supervision für Sie zu? [To what extent do the following statements regarding the importance of supervision apply to you?] *

	trifft gar nicht zu [not applicable]	trifft eher nicht zu [rather not applicable]	trifft teilweise zu [partly applicable]	trifft eher zu [applicable]	trifft sehr zu [very applicable]
Die Supervision war für mich persönlich eine Bereicherung bezüglich meiner Selbst-wahrnehmung und -reflexion. [The supervision was personally enriching for me in terms of my self-awareness and self-reflection.]					
Die Supervision war für mich persönlich eine Bereicherung hinsichtlich meines Wissens über Fälle und Themen betreffend die Dolmetscheinsätze. [The supervision was personally enriching for me in terms of my knowledge about cases and topics related to interpreting assignments.]					
Der zeitliche Aufwand der Supervision hat sich gelohnt. [The time spent on the supervision was worth it.]					
Ich würde die Supervision weiterempfehlen. [I would recommend the supervision to others.]					

* Analyzes to these question that were not relevant to the research questions can be requested from the authors if needed.

Wie sehr treffen die folgenden Aussagen bezüglich Ihrer Erfahrungen in der Supervision auf Sie zu? [To what extent do the following statements regarding your experiences in supervision apply to you?]*

	trifft gar nicht zu [not applicable]	trifft eher nicht zu [rather not applicable]	trifft teilweise zu [partly applicable]	trifft eher zu [applicable]	trifft sehr zu [very applicable]
Meine Selbst- und Fremdwahrnehmung hat sich verbessert. [My introspection and perception of others have improved.]					
Ich konnte meine Probleme/Belastungen offen ansprechen. [I was able to openly address my issues/concerns.]					
Meine berufliche Rolle ist mir klarer geworden. [My professional role has become clearer to me.]					
Ich fühle mich im Umgang mit den AnwenderInnen und KundInnen sicherer. [I feel more confident in interacting with clients and customers.]					
Ich habe mehr Empathie für die AnwenderInnen/KundInnen entwickelt. [I have developed a greater sense of empathy for clients and customers.]					
Ich kann besser mit emotional belastenden Dolmetsch-gesprächen umgehen. [I have improved in dealing with emotionally					

challenging interpreting sessions.]					
Ich lernte mit konfliktreichen Gesprächen umzugehen. [I have learned to handle conflict-ridden conversations.]					
Ich habe neue Sichtweisen bekommen. [I have gained new perspectives.]					
Ich kann besser mit meinen Grenzen umgehen. [I have improved in managing my boundaries.]					
Ich kann mich in meiner Freizeit besser von der Arbeit abgrenzen. [I can better detach myself from work during my leisure time.]					
Ich kann besser mit Stress und Zeitdruck umgehen. [I have become better at handling stress and time pressure.]					

Wie sehr treffen die folgenden Aussagen bezüglich Ihrer Erfahrungen in der GRUPPENsupervision auf Sie zu? [To what extent do the following statements regarding your experiences in GROUP supervision apply to you?]*

	trifft gar nicht zu [not applicable]	trifft eher nicht zu [rather not applicable]	trifft teilweise zu [partly applicable]	trifft eher zu [applicable]	trifft sehr zu [very applicable]
Die Gruppenatmosphäre in den Supervisionen unterstützte die gemeinsame Reflexion. [The group atmosphere in the supervision sessions					

* Analyzes to these question that were not relevant to the research questions can be requested from the authors if needed.

supported collective reflection.]					
Die Themen und Inhalte in den Gruppensupervision haben mich betroffen. [The topics and content in the group supervision sessions were relevant to me.]					
Ich konnte mich an den Gesprächen in der Gruppensupervision beteiligen. [I was able to participate in the discussions during the group supervision sessions.]					
Ich profitierte von Fällen bzw. Themen, welche andere DolmetscherInnen eingebracht haben. [I benefited from the cases or topics that other interpreters brought up.]					

Diese Themen, Inhalte und/oder Methoden in den Gruppensupervisionen waren für mich besonders hilfreich [These topics, content, and/or methods in the group supervision sessions were particularly helpful to me:]: *

Was würden Sie sich in Zukunft von der Supervision wünschen? [What would you wish for in future supervision sessions?]*

Nachfolgend noch einige Fragen zu Ihrer Person [Here are a few more questions about yourself]:

Ihr Geschlecht [Your gender]

Weiblich [female]

Männlich [male]

Anderes, und zwar: [Other, specifically:]

Ihr Alter [Your age]: ______ Jahre [years]

Ihre Muttersprache [Your mother tongue]: _______________*

Was ist Ihre höchste abgeschlossene Schulbildung? [What is your highest completed level of education?]*

Pflichtschule [Mandatory school education]

Matura/Abitur [High school diploma]

Bachelorstudium [Bachelor's degree]

Masterstudium bzw. Magister-/Diplomstudium [Master's degree or equivalent]

Doktorat/PHD [Doctorate/PhD]

Welche Qualifikation haben Sie für Ihre Tätigkeit als DolmetscherIn? (Mehrfachnennungen sind möglich) [What qualifications do you have for your work as an interpreter? (Multiple answers are possible)]*

Translationswissenschaftliches Studium [Degree in Translation Studies]

Beeid(ig)ung/gerichtliche Zertifizierung [Sworn/court certification]

Staatliche Prüfung als DolmetscherIn [State examination as an interpreter]

Universitäre Fort- und Weiterbildungen im Dolmetschen [University-based continuing education in interpreting]

Außeruniversitäre Fort- und Weiterbildungen im Dolmetschen (z.B. angeboten von Behörden, Vereinen, NGOs, etc.) [Non-university-based continuing education in interpreting (e.g., offered by authorities, associations, NGOs, etc.)]

* Analyzes to these question that were not relevant to the research questions can be requested from the authors if needed.
